# A Group Decision Making Approach Considering Self-Confidence Behaviors and Its Application in Environmental Pollution Emergency Management

**DOI:** 10.3390/ijerph16030385

**Published:** 2019-01-29

**Authors:** Xia Liu, Yejun Xu, Yao Ge, Weike Zhang, Francisco Herrera

**Affiliations:** 1 Business School, Hohai University, Nanjing 211100, China; liuxia@hhu.edu.cn (X.L.); xuyejohn@163.com (Y.X.); 2 Andalusian Research Institute in Data Science and Computational Intelligence (DaSCI), University of Granada, 18071 Granada, Spain; zhangwk@correo.ugr.es (W.Z.); herrera@decsai.ugr.es (F.H.); 3 School of Economics, Sichuan University, Chengdu 610064, China; 4 Faculty of Computing and Information Technology, King Abdulaziz University, Jeddah 21589, Saudi Arabia

**Keywords:** group decision making, self-confidence, decision weight, environmental pollution emergency management, score function

## Abstract

Self-confidence as one of the human psychological behaviors has important influence on emergency management decision making, which has been ignored in existing methods. To fill this gap, we dedicate to design a group decision making approach considering self-confidence behaviors and apply it to the environmental pollution emergency management. In the proposed method, the self-confident fuzzy preference relations are utilized to express experts’ evaluations. This new type of preference relations allow experts to express multiple self-confidence levels when providing their evaluations, which can deal with the self-confidence of them well. To apply the proposed group decision making method to environmental pollution emergency management, a novel determination of the decision weights of experts is given combining the subjective and objective weights. The subjective weight can be directly assigned by organizer, while the objective weight is determined by the self-confidence degree of experts on their evaluations. Afterwards, by utilizing the weighted averaging operator, the individuals’ evaluations can be aggregated into a collective one. To do that, some operational laws for self-confident fuzzy preference relations are introduced. And then, a self-confidence score function is designed to get the best solution for environmental pollution emergency management. Finally, some analyses and discussions show that the proposed method is feasible and effective.

## 1. Introduction

Group decision making (GDM) refers to a condition whereby a group of experts (decision makers) are involved in a decision process, provide their evaluations regarding the provided alternatives, and select an optimal decision by the aggregation of their opinions [1]. Generally, in GDM problems, preference relations are the most common representation of information, because it is a useful tool in modeling decision processes. The main advantage of preference relations is that individuals can focus exclusively on two alternatives at a time, which facilitating the expression of their opinions [2,3], and then making them more accurate than non-pairwise methods [4]. To date, many different types of preference relations have been proposed and widely used in decision making problems [5,6,7,8,9,10,11].

As we all know, real GDM situations involve not only the fields of mathematics but also the human psychological behaviors [12]. Self-confidence as one of the human psychological behaviors has important influence on decision making [13,14,15,16,17]. Hence, it would be of great importance to design a GDM approach considering self-confidence behavior. To do so, Liu, et al. [18] introduced a new type of preference relation called self-confident fuzzy preference relation (SC-FPR), which allows experts to express multiple self-confidence levels when providing their evaluations. In an SC-FPR, the elements are composed of two components, the former represents the preference degree between pairs of alternatives, and the latter denotes the self-confidence level associated to the first component. Obviously, the second element indicates a psychological expression for personal self-estimation.

**Figure 1:**
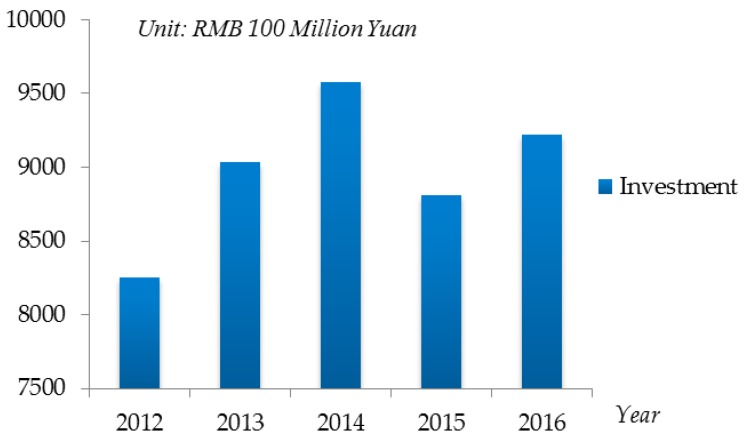
The per year investment in environmental pollution emergency management from 2012 to 2016, China.

Nowadays, due to a lack of attention to the negative effects of highly developed industries, environmental pollution problems, which are one of the three major crises in the world, are emerging one after another. Specially, in China, with the rapid development of economic and technology, the environmental pollution turns into a high-risk period, a series of environmental pollution events have occurred in recent years. For instance, the dead fish incident in Baiyangdian in Hebei Province in 2006 [19], the water pollution in Taihu Lake in 2007 [20], the cadmium pollution in Longjiang River in Guangxi Province in 2012 [21], the major water pollution incident in the Songhua River in 2015 [22], the pig deaths in Songjiang River in Shanghai in 2017 [23] and so on. In order to improve the efficiency of environmental pollution emergency management, as well as to reduce the risk and damage of environmental pollution, the Chinese government is also increasingly investing in environmental protection. Figure 1 shows the per year investment in environmental pollution emergency management in China from 2012 to 2016.

Although the Chinese government and the public are now paying great attention to environmental protection, the environmental pollution problem has not fundamentally been resolved yet. Table 1 shows the number of the environmental emergencies occurred per year from 2012 to 2016 in China. Clearly, from Table 1, we can observe that serious environmental emergencies are still common in China. In other words, the environmental pollution emergency management is still a hotspot that cannot be ignored in China.

Up to now, there are several studies related to environmental pollution emergency management. Such as, Yang [24] proposed an environmental emergency response plan for EHS management of mega-construction projects. Shao, et al. [25] presented an integrated environmental risk assessment, as well as designed a whole process management system in chemical industry parks. Zhang and Wang [26] suggested that strengthening the quality management is the most important role of sudden environmental pollution emergency monitoring. In addition, from the perspective of the sustainability and resilience, Marchese, et al. [27] reviewed the integrated use of sustainability and resilience in an environmental management context. Cao, et al. [28] conducted an analytical study of environmental incidents from 2006 to 2015 in China, which has useful implications for policy-making and environmental management. Based on the principle of implementing green policy and community participation, Ayeni [29] discussed the environmental policies for emergency management and public safety. Rämö, et al. [30] made an environmental risk assessment of pesticides in the River Madre de Dios, Costa Rica using PERPEST (predict the ecological risks of pesticides), SSD (species sensitivity distributions), and msPAF (multi-substance potentially affected fraction) models and so on. Through the review of the above literature, it reveals that environmental pollution problem is still a hotpot of the sustainable development of human beings. Meanwhile, all these existing methods have made an important progress in improving the efficiency of environmental pollution emergency management.

As far as we know, one of the most crucial problems concerning the environmental pollution emergency management is to choose a reasonable alternative to reduce the risk and probability of pollution. Thus, in some cases, the environmental pollution emergency management actually can be seen as a GDM problem. However, according to the above literature reviewed regarding the environmental pollution emergency management, we find that from the perspective of GDM, and take the self-confidence behaviors of experts into account, to discuss the environmental pollution emergency management is still a challenge.

To fill the gap mentioned above, we devote this paper to discussing a GDM approach considering self-confidence behaviors and apply it to environmental pollution emergency management. In the proposed method, the SC-FPRs are utilized to express experts’ evaluations. Subsequently, a novel determination of the decision weights of experts is given to apply the proposed GDM method to environmental pollution emergency management. Furthermore, by utilizing the weighted averaging (WA) operator, the individuals’ evaluations can be aggregated into a collective one. To do that, some new operational laws for SC-FPR are introduced. And then, a self-confidence score function (SCS) is designed to get the best solution for environmental pollution emergency management. The main novelties and contributions of this paper are listed as follows:

1. Experts’ self-confidence levels are taken into account in GDM problems. That is, experts are allowed use SC-FPRs to express their evaluations, which can deal with their self-confidence psychological behaviors well. Meanwhile, some new operation laws for SC-FPRs are proposed to apply to environmental pollution emergency management.
2. A novel determination of the decision weights of experts is given combining the subjective and objective weights. On one hand, the subjective weight can be directly assigned by organizer. On the other hand, the objective weight is determined by the self-confidence degree (SCD) of experts on their evaluations.
3. An SCD is presented to measure the overall self-confidence levels of experts on their evaluations, as well as to be utilized to assign their objective weights in environmental pollution emergency management.
4. An SCS function for SC-FPRs is designed to select the best alternative(s) in environmental pollution emergency management. We rank alternatives by computing the *SCSs* of the collective evaluations. And then, the best alternative is the one with the highest *SCS*.

The effectiveness of the research in this paper is demonstrated by a case of study of environmental pollution emergency management. Moreover, some comparative analyses and discussions are provided to validity the self-confidence of experts’ impact on final decision. From the results, it concludes that the self-confidence levels of experts have important influence on the alternative ranking in environmental pollution emergency management.

The rest of this paper is organized as follows: in Section 2, we review some preliminaries regarding the 2-tuple linguistic ordinal scale model and SC-FPRs. In Section 3, a determination of the decision weights of experts and the GDM approach considering self-confidence are presented. Section 4 applies the proposed GDM method to resolve an environmental pollution emergency management. Subsequently, some analyses and discussions are shown in Section 5. Finally, the conclusions are summarized in Section 6.

## 2. Preliminaries

This section reviews some related knowledge regarding the 2-tuple linguistic ordinal scale model, and SC-FPRs. For simplicity, some symbol descriptions which used in the whole paper are shown in Appendix A.

### 2.1. 2-Tuple Linguistic Ordinal Scale Model

To carry out ordinal computing with words when dealing with the linguistic self-confidence in GDM problems, the 2-tuple linguistic ordinal scale model is reviewed as follows.

Let $S=\{s_{i}|i=0,1,\ldots ,g\}$ be a linguistic term set. The term $s_{i}$ denotes a possible value of a linguistic variable, and the order on set *S* is assumed that $s_{i}>s_{j}$ if and only if i>j. Then, the concept of 2-tuple fuzzy linguistic model is given below:

**Definition** **1** **[31].***Let*β∈[0,g]*be a number in the granularity interval of the linguistic term set S. Let*i=round(β)*and*α=β−i*be two values such that*i∈[0,g]*, and*α∈[−0.5,0.5). *Then*α*is called a symbolic translation, and the round is the usual round operation.*

Herrera and Martínez [31] developed a linguistic representation model which represents the linguistic information by means of 2-tuples $\left(s_{i},\alpha \right)$, $s_{i}\in S$ and α∈[−0.5,0.5). Obviously, the 2-tuple linguistic model defines a function to make transformation between linguistic 2-tuples and numerical values.

**Definition** **2** **[31].** *Let S be a linguistic term set with the granularity interval* [0,g] *. The 2-tuple that expresses the equivalent information to* β∈[0,g] *is obtained with the following function:* Δ:[0,g]→S×[−0.5,0.5) *, where:*

$$\Delta (\beta )=(s_{i},\alpha ),\;\mathrm{with}\;\{\begin{array}{cc} s_{i}, & i=round(\beta )\\ \alpha =\beta -i, & \alpha \in [-0.5,0.5) \end{array}.$$

Moreover, the Δ represents one to one mapping function. For a linguistic term set *S*, and a 2-tuple $\left(s_{i},\alpha \right)$, there is always an inverse function $\Delta^{-}$ can from a 2-tuple returns its equivalent numerical value β∈[0,g]:

$$\Delta^{-}:S\times [-0.5,0.5)\to [0,g];$$

$$\Delta^{-}(s_{i},\alpha )=i+\alpha =\beta .$$

Clearly, the conversion of a linguistic term into a linguistic 2-tuple consist of adding a value zero as symbolic translation $s_{i}\in S\Rightarrow (s_{i},0)$, i.e., $\Delta^{-}(s_{i},0)=\Delta^{-}(s_{i})$. Additionally, some computations and operators were presented to deal with 2-tuple linguistic information in [31,32,33] as follows:

(1) 2-tuples comparison operator: Let $\left(s_{v},\alpha \right)$ and $\left(s_{l},\gamma \right)$ be two 2-tuples, then:
  - if v<l, then $\left(s_{v},\alpha \right)$ is smaller than $\left(s_{l},\gamma \right)$;
  - if v=l, then
    (a) if α=γ, then $\left(s_{v},\alpha \right)$, $\left(s_{l},\gamma \right)$ represents the same information;
    (b) if α<γ, then $\left(s_{v},\alpha \right)$ is smaller than $\left(s_{l},\gamma \right)$;
(2) A 2-tuple negation operator:
  $$Neg(s_{i},\alpha )=\Delta (g-\Delta^{-}(s_{i},\alpha )).$$

### 2.2. Self-Confident Fuzzy Preference Relations

As we all know, real GDM problems involve not only the fields of mathematics but also human psychological behavior. Self-confidence as one of the human psychological traits that has an important influence on decision making [13,14,15,16,17]. Hence, it would be of great importance to take the self-confidence behaviors of experts into account in GDM problems. To do so, a new preference relation called SC-FPR introduced by [18], which can be utilized to deal with the self-confidence of expert well.

Suppose a linguistic term $S^{SL}=\{s_{i}|i=0,1,\ldots ,g\}$ is used to characterize experts’ self-confidence over their evaluations. Without loss of generality, this paper assumes that experts use a nine linguistic-term set $S^{SL}=\{s_{0},s_{1},\ldots ,s_{8}\}$ to express their self-confidence levels. The detailed information of is shown in Table 2.

The definition of SC-FPR introduced by [18] is given below:

**Definition** **3** **[18].***A matrix*$P={\left(p_{ij},l_{ij}\right)}_{n\times n}$*is called an SC-FPR, where the first component*$p_{ij}\in [0,1]$*represents the preference degree of the alternative*$x_{i}$*over*$x_{j}$*. The second one*$l_{ij}\in S^{SL}$*denotes the self-confidence level associated to the first element*$p_{ij}$*. The following conditions are assumed:*$p_{ij}+p_{ji}=1$*,*$p_{ii}=0.5$*,*$l_{ij}=l_{ji}$*and*$l_{ii}=s_{g}$*for*∀i,j=1,2,…,n.

*Note*: As far as we know, the Z-number which introduced by Zadeh [34] is consists of an ordered pair of fuzzy numbers. The first element denotes the constraint on the values on the real-valued uncertain variable, and the second represents the measure of reliability of the first element. Therefore, in some cases, the elements in an SC-FPR also can be seen as a Z-number.

**Example** **1.** *Assume that an expert assesses four alternatives and provides an SC-FPR as follows*:

$$P=\left(\begin{array}{cccc} \left(0.5,s_{8}\right) & \left(0.4,s_{5}\right) & \left(0.2,s_{7}\right) & \left(0.7,s_{8}\right)\\ \left(0.6,s_{5}\right) & \left(0.5,s_{8}\right) & \left(0.8,s_{6}\right) & \left(0.9,s_{4}\right)\\ \left(0.8,s_{7}\right) & \left(0.2,s_{6}\right) & \left(0.5,s_{8}\right) & \left(0.6,s_{5}\right)\\ \left(0.3,s_{8}\right) & \left(0.1,s_{4}\right) & \left(0.4,s_{5}\right) & \left(0.5,s_{8}\right) \end{array}\right).$$

In the *P*, $p_{12}=0.4$ means the preference degree of the alternative $x_{1}$ over the alternative $x_{2}$ is 0.4, and the $l_{12}=s_{5}$ shows the expert’s self-confidence level associated to $p_{12}$ is $s_{5}$. That is, the expert is slightly high self-confident in her/his evaluation. In addition, all the other elements in *P* can be explained similarly.

In order to effectively aggregate the information in GDM problems, and apply the SC-FPRs to environmental pollution emergency management, based on the transitivity rule, some new operational laws of 2-tuples in SC-FPR are defined by [35] as follows.

**Definition** **4** **[35].** *Assume* $\left(p_{i},l_{i}\right)$ *,* $\left(p_{z},l_{z}\right)$ *are two 2-tuples,* $p_{i}$ *,* $p_{z}$ *are the fuzzy preference values, and* $l_{i}$ *,* $l_{z}$ *are corresponding self-confidence levels, where* $l_{i},l_{z}\in S^{SL}$ *,* λ∈[0,1] *. Then, we have the following operations:*

*(1)*  $\left(p_{i},l_{i})+(p_{z},l_{z})=(p_{i}+p_{z},\min\{l_{i},l_{z}\}\right)$;
*(2)*  $\left(p_{i},l_{i})-(p_{z},l_{z})=(p_{i}-p_{z},\min\{l_{i},l_{z}\}\right)$;
*(3)*  $\left(p_{z},l_{z})-\lambda =(p_{z}-\lambda ,l_{z}\right)$;
*(4)*  ${\left(p_{z},l_{z}\right)}^{\lambda }=({\left(p_{z}\right)}^{\lambda },l_{z})$;
*(5)*  $\lambda (p_{z},l_{z})=(\lambda p_{z},l_{z})$.

## 3. A GDM Approach Considering Self-Confidence Behaviors

In this section, a GDM approach considering self-confidence behaviors is proposed. Firstly, in Section 3.1, a novel determination of the decision weights of experts considering self-confidence level is presented. Afterwards, the detailed decision processes for GDM considering self-confidence are presented in Section 3.2.

### 3.1. Determine the Decision Weight of Expert Considering Self-Confidence

One of the necessary stages in GDM analysis is to combine the individuals’ evaluations and weights to form a collective evaluation. Thus, the determination of the decision weight of expert is of great importance.

Generally, in the most existing methods, all experts involved in decision making are directly assigned equal weights by the organizer. That is, each expert is assumed to play an equally important role in GDM. Nevertheless, due to experts have different knowledge or experience, they may express different self-confidence levels when providing their evaluations. The self-confidence usually indicates the self-recognition of expert on her/his evaluation, the higher the self-confidence will imply the more the knowledge or experience of expert for GDM problems. Hence, the self-confidence levels of experts should be considered on the determination of the decision weight of expert. To do so, a novel determination of the decision weights of experts considering self-confidence levels is presented in this research. It mainly contains the following three stages:

*Stage 1*. The organizer directly assigns the subjective weight for each expert denoted as $w^{sub}=(w_{1}^{sub},w_{2}^{sub},\ldots ,w_{m}^{sub})$, where $w_{k}^{sub}$ represents the subjective weight of expert $e_{k}$, such that $\sum_{k=1}^{m}w_{k}^{sub}=1$ and $w_{k}^{sub}\in [0,1]$, k=1,2,…,m. Considering the fairness among experts, the $w_{k}^{sub}$ can be determined by the number of participators in decision making as follows:
  (1) $$w_{k}^{sub}=\frac{1}{k},\;k=1,2,\ldots m$$
*Stage 2*. To determine the objective weights of experts based on the *SCDs* in their evaluations. The detailed approach is described below:

Actually, as per the SC-FPR $P={\left(p_{ij},l_{ij}\right)}_{n\times n}$ given by an expert, it can be seen as a combination of FPR $\tilde{P}={\left({\tilde{p}}_{ij}\right)}_{n\times n}$ and a self-confidence matrix $\tilde{L}={\left({\tilde{l}}_{ij}\right)}_{n\times n}$, where ${\tilde{p}}_{ij}=p_{ij}$ and ${\tilde{l}}_{ij}=l_{ij}$ for ∀i,j=1,2,…,n. As aforementioned, the higher the self-confidence of the experts on their evaluations, the more the knowledge or experience of them for decision making problems will be. Based on this hypothesis, we propose to measure the *SCD* of expert by measuring the deviation level between the self-confidence matrix of expert and the maximal self-confidence matrix. To do so, the self-confidence deviation level (*SCDL*) between the self-confidence matrix of SC-FPR given by expert and the maximal self-confidence matrix is defined by:

**Definition** **5.** *Let* $P_{k}={\left(p_{ij,k},l_{ij,k}\right)}_{n\times n}$ *be an SC-FPR given by an expert* $e_{k}$ *, and* ${\tilde{L}}_{k}={\left({\tilde{l}}_{ij,k}\right)}_{n\times n}$ *be the corresponding self-confidence matrix of the expert* $e_{k}$ *. Let* $L={\left(s_{g}\right)}_{n\times n}$ *be the maximal self-confidence matrix, where* ${\tilde{l}}_{ij,k},s_{g}\in S^{SL}$ *. Then, the SCDL of* $e_{k}$ *(* k={1,2,…m} *) is defined as:*

(2) $$SCDL(e_{k})=\frac{2}{n(n-1)}\sum_{i=1}^{n-1}\sum_{j=i+1}^{n}d({\tilde{L}}_{k},L)=\frac{2}{n(n-1)}\sum_{i=1}^{n-1}\sum_{j=i+1}^{n}\frac{\left|\Delta^{-}({\tilde{l}}_{ij,k})-\Delta^{-}(s_{g})\right|}{g}$$

*Note*: Obviously, the $SCDL(e_{k})$ has the following characteristics:

(1) $SCDL(e_{k})\in [0,1]$;
(2) if $SCDL(e_{k})=0$, it means that the expert $e_{k}$ is completely self-confident in all of her/his evaluations.

Afterwards, the *SCD* of the expert $e_{k}$ is given below:

(3) $$SCD(e_{k})=1-SCDL(e_{k})$$

Similarly, we have $SCD(e_{k})\in [0,1]$. The higher the value of the $SCD(e_{k})$, the more the self-confidence levels of the expert $e_{k}$ in her/his evaluations will be.

As mentioned above, the *SCDs* can reflect the experts’ knowledge, abilities or experiences. The higher the value of *SCD* of expert, the more the reliable of her/his assessment information will be. Moreover, the higher the quality and efficiency of emergency management will be. Thus, the expert who has most self-confident should be assigned larger weight in GDM problems. Let $w^{obj}=(w_{1}^{obj},w_{2}^{obj},\ldots ,w_{m}^{obj})$ be the objective weight vectors of experts, where $w_{k}^{obj}$ represents the objective weight of the expert $e_{k}$ (k=1,2,…,m), the $w_{k}^{obj}$ can be computed by:

(4) $$w_{k}^{obj}=\frac{SCD(e_{k})}{\sum_{k=1}^{m}SCD(e_{k})}$$

where $\sum_{k=1}^{m}w_{k}^{obj}=1$ and $w_{k}^{obj}\in [0,1]$, k=1,2,…,m.

*Stage 3*. Based on the above analysis, let $w=(w_{1},w_{2},\ldots ,w_{k})$ be the weight vector of experts, the $w_{k}$ can be determined by combing the subjective weight $w_{k}^{sub}$ and the objective weight $w_{k}^{obj}$ as follows:
  (5) $$w_{k}=\mu w_{k}^{sub}+\tau w_{k}^{obj},\;k=1,2,\ldots ,m$$
  where μ and τ are parameters to control the weight between $w_{k}^{sub}$ and $w_{k}^{obj}$ of expert $e_{k}$, μ,τ∈[0,1] and μ+τ=1.

*Note*: As per Equation (5), we have the following two conclusions:

(1) if μ=1, i.e., τ=0, it indicates that the weight of expert does not consider the objective weight. In other words, the self-confidence of expert is not taken into account.
(2) if μ=0, i.e., τ=1, it represents that the subjective weight of expert is not considered.

Without loss of generality, this paper assumes that the subjective weight and the objective weight are equally important on determination of the decision weight of expert. Thus, we have μ=τ=0.5.

### 3.2. Detailed Decision Processes for GDM Considering Self-Confidence

After the decision weights of experts are obtained, a collective evaluation can be computed by the WA operator. Let $P_{c}={\left(p_{ij,c},l_{ij,c}\right)}_{n\times n}$ be the collective evaluation, where:

(6) $$(p_{ij,c},l_{ij,c})=\sum_{k=1}^{m}w_{k}(p_{ij,k},l_{ij,k})$$

According to the Definition 4 and Equation (6), the collective preference information can be computed. Subsequently, the GDM turns to a selection process. That is, we need to transform the collective preference information of the alternatives into a collective ranking, and then to get the best alternative for the GDM problem. To do so, for a collective SC-FPR, we propose to choose the best alternative by computing the *SCS* of each alternative. The alternative with the highest *SCS* should be chosen as the optimal alternative. The *SCS* function of SC-FPR is defined as:

**Definition** **6.** *Let* $X=\{x_{1},\ldots ,x_{n}\}$ *be an alternative set, and* $P_{c}={\left(p_{ij,c},l_{ij,c}\right)}_{n\times n}$ *be the collective SC-FPR of GDM, then the SCS of each alternative can be computed as follows:*

(7) $$SCS(x_{i})=\frac{1}{n}\sum_{j=1}^{n}\left(p_{ij,c}\times \Delta^{-}(l_{ij,c}\right)),\;i=1,2,\ldots ,n$$

*Note*: As per Definition 6, the higher the value of the $SCS(x_{i})$, the more expert self-confident of the alternative $x_{i}$ will be. That is, if we have $SCS(x_{i})>SCS(x_{j})$, then $x_{i}≻x_{j}$, $x_{i},x_{j}\in X$.

In addition, the detailed GDM approach considering self-confidence is depicted in Algorithm 1.


**Algorithm 1.**
*The GDM approach considering self-confidence.*
*Step 1***.** Suppose that there is a set of alternatives $X=\{x_{1},x_{2},\ldots ,x_{n}\}$. Some experts are invited to take part in the decision making, and $E=\{e_{1},e_{2},\ldots ,e_{m}\}$ is a set of experts. All of the experts make the pairwise comparison of the alternatives in *X*, and then use SC-FPRs to express their evaluations, denoted as $P_{k}={\left(p_{ij,k},l_{ij,k}\right)}_{n\times n}$ (k=1,2,…,m). Go to Step 2. *Step 2***.** Utilize Equations (1) and (4) to compute the $w_{k}^{sub}$ and $w_{k}^{obj}$ of expert $e_{k}$, respectively. Subsequently, the decision weight $w_{k}$ of expert $e_{k}$ can be calculated by Equation (5). Go to Step 3. *Step 3***.** Compute the collective evaluation $P_{c}={\left(p_{ij,c},l_{ij,c}\right)}_{n\times n}$ by Equation (6). Go to Step 4. *Step 4***.** Calculate the *SCS* of each alternative in collective evaluation by Equation (7). And then, the optimal selection can be obtained. *Step 5***.** End.

## 4. Case Study: An Environmental Pollution Emergency Management

Self-confidence as one of the human psychological behaviors has a great influence on emergency management decision making, which has been ignored in most existing studies. To fill this gap, in this section, we apply the proposed GDM method considering self-confidence to an environmental pollution emergency management. In Section 4.1, the environmental pollution emergency management description is given. An application of the proposed GDM method considering self-confidence to environmental pollution emergency management is shown in Section 4.2.

### 4.1. Environmental Pollution Emergency Management Description

With the rapid development of economic and technology, the environmental pollution in China turns into a high-risk period, a series of environmental pollution events have occurred recently. In order to improve the efficiency of the management of environmental pollution emergency, as well as to reduce the risk of environmental pollution, the city *Q* decided to make an environmental pollution emergency plan.

Combined with the policy of the national environmental protection department and the specific of the city *Q*, the organizer provides four environmental pollution emergency plans (possible solutions) $x_{1},x_{2},x_{3},x_{4}$ to be chosen. Subsequently, four experts $e_{1},e_{2},e_{3},e_{4}$ from the Environmental Protection Department, Emergency Management Department, and the Research Institute of Universities are invited to participate in the decision making. Considering the quality and efficiency of final decision, the organizer provides the following four important criteria for the experts:

- Resource allocation;
- The rescue time of environmental pollution emergency;
- The cost of investment;
- Other emergency safeguards.

Based on the above four criteria, each expert is invited to make a pairwise comparison for the provided alternatives, and then uses SC-FPR to express her/his evaluations. Meanwhile, the organizer assigns the equal subjective weight for each expert. In addition, as for the fuzzy preference values in the SC-FPR given by expert, we have the following explanations:

- if $p_{ij,k}=0.5$, it means that expert $e_{k}$ thinks there is indifference between alternatives $x_{i}$ and $x_{j}$, that is, $x_{i}∼x_{j}$.
- if $0.5<p_{ij,k}\leq 1$, it means that expert $e_{k}$ thinks alternative $x_{i}$ is preferred to alternative $x_{j}$, that is, $x_{i}≻x_{j}$. Specially, if $p_{ij,k}=1$, it indicates that expert $e_{k}$ thinks alternative *x_i_* is definitely preferred to alternative *x_j_*.
- if $0\leq p_{ij,k}<0.5$, it means that expert $e_{k}$ thinks alternative $x_{j}$ is preferred to alternative $x_{i}$, that is, $x_{j}≻x_{i}$. Meanwhile, the smaller $p_{ij,k}$ the stronger the preference of $x_{j}$ over $x_{i}$.

### 4.2. Application of the Proposed GDM Approach Considering Self-Confidence

In this section, Algorithm 1 is utilized to select the best solution for the environmental pollution emergency management. The detailed steps are as follows:

*Step 1*. Suppose that the SC-FPRs given by these four experts are:

$$\begin{array}{c} P_{1}=\left(\begin{array}{cccc} \left(0.5,s_{8}\right) & \left(0.1,s_{5}\right) & \left(0.6,s_{7}\right) & \left(0.7,s_{4}\right)\\ \left(0.9,s_{5}\right) & \left(0.5,s_{8}\right) & \left(0.8,s_{6}\right) & \left(0.6,s_{4}\right)\\ \left(0.4,s_{7}\right) & \left(0.2,s_{6}\right) & \left(0.5,s_{8}\right) & \left(0.6,s_{5}\right)\\ \left(0.3,s_{4}\right) & \left(0.4,s_{4}\right) & \left(0.4,s_{5}\right) & \left(0.5,s_{8}\right) \end{array}\right),\\ P_{2}=\left(\begin{array}{cccc} \left(0.5,s_{8}\right) & \left(0.6,s_{3}\right) & \left(0.8,s_{5}\right) & \left(0.2,s_{3}\right)\\ \left(0.4,s_{3}\right) & \left(0.5,s_{8}\right) & \left(0.6,s_{4}\right) & \left(0.7,s_{6}\right)\\ \left(0.2,s_{5}\right) & \left(0.4,s_{4}\right) & \left(0.5,s_{8}\right) & \left(0.4,s_{3}\right)\\ \left(0.8,s_{3}\right) & \left(0.3,s_{6}\right) & \left(0.6,s_{3}\right) & \left(0.5,s_{8}\right) \end{array}\right), \end{array}$$

$$\begin{array}{c} P_{3}=(\begin{array}{cccc} \left(0.5,s_{8}\right) & \left(0.3,s_{6}\right) & \left(0.4,s_{5}\right) & \left(0.7,s_{4}\right)\\ \left(0.7,s_{6}\right) & \left(0.5,s_{8}\right) & \left(0.2,s_{6}\right) & \left(0.4,s_{3}\right)\\ \left(0.6,s_{5}\right) & \left(0.8,s_{6}\right) & \left(0.5,s_{8}\right) & \left(0.9,s_{2}\right)\\ \left(0.3,s_{4}\right) & \left(0.6,s_{3}\right) & \left(0.1,s_{2}\right) & \left(0.5,s_{8}\right) \end{array}),\\ P_{4}=\left(\begin{array}{cccc} \left(0.5,s_{8}\right) & \left(0.4,s_{5}\right) & \left(0.2,s_{6}\right) & \left(0.1,s_{5}\right)\\ \left(0.6,s_{5}\right) & \left(0.5,s_{8}\right) & \left(0.6,s_{6}\right) & \left(0.5,s_{1}\right)\\ \left(0.8,s_{6}\right) & \left(0.4,s_{6}\right) & \left(0.5,s_{8}\right) & \left(0.3,s_{7}\right)\\ \left(0.9,s_{5}\right) & \left(0.5,s_{1}\right) & \left(0.7,s_{7}\right) & \left(0.5,s_{8}\right) \end{array}\right). \end{array}$$

*Step 2*. By Equation (1), we get the subjective weights of experts $e_{k}$ (k=1,2,3,4):

$$w_{1}^{sub}=1/4,\;w_{2}^{sub}=1/4,\;w_{3}^{sub}=1/4,\;w_{4}^{sub}=1/4.$$

Afterwards, based on Definition 5, we have the self-confidence matrix ${\tilde{L}}_{k}={\left({\tilde{l}}_{ij,k}\right)}_{n\times n}$ of experts $e_{k}$ (k=1,2,3,4):

$${\tilde{L}}_{1}=\left(\begin{array}{cccc} s_{8} & s_{5} & s_{7} & s_{4}\\ s_{5} & s_{8} & s_{6} & s_{4}\\ s_{7} & s_{6} & s_{8} & s_{5}\\ s_{4} & s_{4} & s_{5} & s_{8} \end{array}\right),\;{\tilde{L}}_{2}=\left(\begin{array}{cccc} s_{8} & s_{3} & s_{5} & s_{3}\\ s_{3} & s_{8} & s_{4} & s_{6}\\ s_{5} & s_{4} & s_{8} & s_{3}\\ s_{3} & s_{6} & s_{3} & s_{8} \end{array}\right),$$

$${\tilde{L}}_{3}=\left(\begin{array}{cccc} s_{8} & s_{6} & s_{5} & s_{4}\\ s_{6} & s_{8} & s_{6} & s_{3}\\ s_{5} & s_{6} & s_{8} & s_{2}\\ s_{4} & s_{3} & s_{2} & s_{8} \end{array}\right),\;{\tilde{L}}_{4}=\left(\begin{array}{cccc} s_{8} & s_{5} & s_{6} & s_{5}\\ s_{5} & s_{8} & s_{6} & s_{1}\\ s_{6} & s_{6} & s_{8} & s_{7}\\ s_{5} & s_{1} & s_{7} & s_{8} \end{array}\right).$$

Compute the $SCDL(e_{k})$ (k=1,2,3,4) by Equation (2). And then, the $SCD(e_{k})$ (k=1,2,3,4) can be obtained by Equation (3). The detailed results are shown in Table 3.

Then, the objective weights of experts $e_{k}$ (k=1,2,3,4) by Equation (4) are:

$$w_{1}^{obj}=0.279,\;w_{2}^{obj}=0.216,\;w_{3}^{obj}=0.234,\;w_{4}^{obj}=0.271.$$

Afterwards, we have the decision weights of experts $e_{k}$ (k=1,2,3,4) by Equation (5) (μ=τ=0.5):

$$w_{1}=0.265,\;w_{2}=0.233,\;w_{3}=0.242,\;w_{4}=0.26.$$

*Step 3*. Compute the collective evaluation $P_{c}={\left(p_{ij,c},l_{ij,c}\right)}_{n\times n}$ by Equation (6):

$$P_{c}=\left(\begin{array}{cccc} \left(0.5,s_{8}\right) & \left(0.33,s_{3}\right) & \left(0.49,s_{5}\right) & \left(0.43,s_{2}\right)\\ \left(0.67,s_{3}\right) & \left(0.5,s_{8}\right) & \left(0.56,s_{4}\right) & \left(0.55,s_{1}\right)\\ \left(0.51,s_{5}\right) & \left(0.44,s_{4}\right) & \left(0.5,s_{8}\right) & \left(0.55,s_{2}\right)\\ \left(0.57,s_{3}\right) & \left(0.45,s_{1}\right) & \left(0.45,s_{2}\right) & \left(0.5,s_{8}\right) \end{array}\right).$$

*Step 4.* By Equation (7), the self-confidence scores $SCS(x_{i})$ (i=1,2,3,4) of all the alternatives are calculated as:

$$SCS(x_{1})=2.184,\;SCS(x_{2})=2.182,$$

$$SCS(x_{3})=2.340,\;SCS(x_{4})=1.752.$$

Thus, the ranking of alternatives is $x_{3}≻x_{1}≻x_{2}≻x_{4}$. Then, the optimal solution is $x_{3}$.

## 5. Analyses and Discussion

In order to further verify the validity of the proposed GDM method in this study, this section gives some comparative analyses and discussions. In Section 5.1, the analysis of the impact of experts’ self-confidence on alternative ranking in GDM is provided. And then, a sensitivity analysis of the experts’ weights is provided in Section 5.2.

### 5.1. The Impact of Experts’ Self-Confidence on Alternative Ranking in GDM

As far as we know, the FPRs denote that experts are fully self-confident of their evaluations. The self-confidence levels related to all fuzzy preference values are the same, that is, $l_{ij}=s_{g}$ for ∀i,j=1,2,…,n. Generally, the self-confidence levels are omitted for notation simplification in FPRs. Thus, the FPRs can be seen a special case of SC-FPRs. As per the case study provided in Section 4, suppose the four experts are fully self-confident of their evaluations. Then, the FPRs, denoted as ${\tilde{P}}_{k}=({\tilde{p}}_{ij,k})$ (k=1,2,3,4) are as follows:

$${\tilde{P}}_{1}=\left(\begin{array}{cccc} 0.5 & 0.1 & 0.6 & 0.7\\ 0.9 & 0.5 & 0.8 & 0.6\\ 0.4 & 0.2 & 0.5 & 0.6\\ 0.3 & 0.4 & 0.4 & 0.5 \end{array}\right),\;{\tilde{P}}_{2}=\left(\begin{array}{cccc} 0.5 & 0.6 & 0.8 & 0.2\\ 0.4 & 0.5 & 0.6 & 0.7\\ 0.2 & 0.4 & 0.5 & 0.4\\ 0.8 & 0.3 & 0.6 & 0.5 \end{array}\right),$$

$${\tilde{P}}_{3}=\left(\begin{array}{cccc} 0.5 & 0.3 & 0.4 & 0.7\\ 0.7 & 0.5 & 0.2 & 0.4\\ 0.6 & 0.8 & 0.5 & 0.9\\ 0.3 & 0.6 & 0.1 & 0.5 \end{array}\right),\;{\tilde{P}}_{4}=\left(\begin{array}{cccc} 0.5 & 0.4 & 0.2 & 0.1\\ 0.6 & 0.5 & 0.6 & 0.5\\ 0.8 & 0.4 & 0.5 & 0.3\\ 0.9 & 0.5 & 0.7 & 0.5 \end{array}\right).$$

Meanwhile, the decision weights of experts $e_{k}$ (k=1,2,3,4) are:

$$w_{1}=0.25,\;w_{2}=0.25,\;w_{3}=0.25,\;w_{4}=0.25.$$

Then, the collective FPR ${\tilde{P}}_{c}=({\tilde{p}}_{ij,c})$ can be obtained by WA operator as:

$${\tilde{P}}_{c}=\left(\begin{array}{cccc} 0.5 & 0.35 & 0.5 & 0.43\\ 0.65 & 0.5 & 0.55 & 0.55\\ 0.5 & 0.4 & 0.5 & 0.55\\ 0.57 & 0.45 & 0.45 & 0.5 \end{array}\right).$$

Afterwards, by Equation (7), the *SCSs* of each alternative can be calculated. And then, we can get the rankings of alternatives of collective evaluations. The detailed results are shown in Table 4.

It is clearly that the rankings of alternatives in Table 4 are different from the results which we have obtained in Section 4.2. Thus, it validates that experts’ self-confidence levels have an important influence on the final decision in GDM problems.

### 5.2. Sensitivity Analysis of the Decision Weight

In this section, a sensitive analysis is conducted to investigate the influence of coefficients μ and τ on the rankings of alternatives. To save the space, the detailed processes are omitted here, and the computation results are directly shown in Table 5.

**Figure 2:**
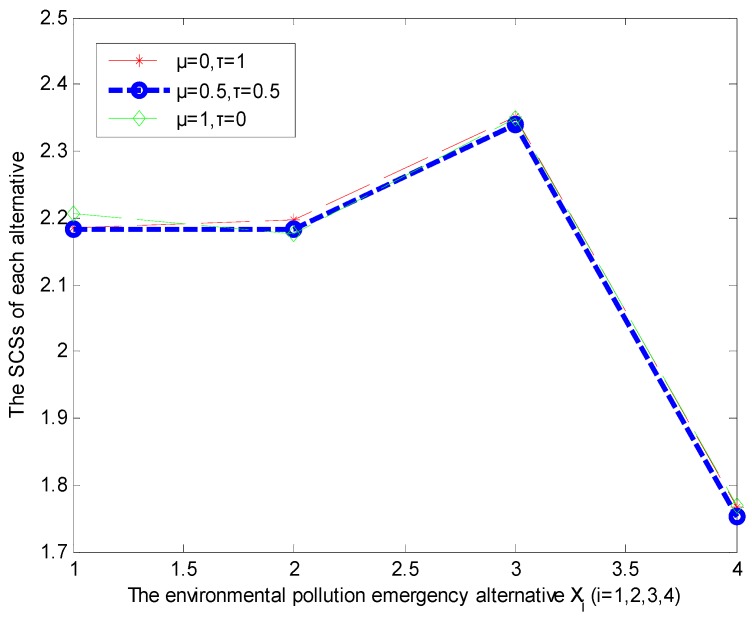
The alternatives ranking with different values of *μ* and *τ*.

From Table 5, it can be seen that the different values of μ and τ have an influence on the ranking of the alternatives. Additionally, Figure 2 shows the several alternatives ranking with different values of μ and τ, where the blue line represents the final alternative rankings. Clearly, from Figure 2 we also reach similar conclusions.

## 6. Conclusions

To improve the quality of emergency management decisions, this paper focuses on the GDM considering self-confidence behaviors and its application in environmental pollution emergency management. The major contributions are summarized below:

(1) Experts are allowed to use SC-FPRs to express their assessment information, so as to deal with the self-confidence psychological behavior well in environmental pollution emergency management. Meanwhile, some new operational laws of 2-tuples in SC-FPR are presented to apply to GDM problems.
(2) A novel determination of the experts’ weights is developed in environmental pollution emergency management. That is, we integrate the subjective weights assigned by the organizer, and the objective weights determined by the experts’ *SCDs* to determine the importance degree of experts in environmental pollution emergency management. An *SCD* is proposed to measure the overall self-confidence levels of experts on their evaluations. Subsequently, the objective weights of experts in environmental pollution emergency management can be assigned by the values of the *SCDs* of experts.
(3) An *SCS* function is designed to obtain the alternatives rankings in environmental pollution emergency management. We compute the values of the *SCSs* for all the alternatives, and then rank them. The best alternative is obtained according to the largest value of *SCS*.

As we all know, the moderate self-confidence of experts is conductive to decision making while overconfident behaviors have a negative impact on the efficiency and quality of final decision(s) [36,37]. Thus, the overconfidence behaviors detection and management of experts in real GDM problems is still an interesting topic for the future. In addition, with the rapid development of science and technology, such as e-democracy [38], social networks [39,40], and public participation [41], more and more decision makers are involved in decision making. This suggests large scale group decision making (LSGDM) will become a research hotspot [42,43,44,45,46,47,48]. Meanwhile, the consensus reaching process (CRP) is an important topic in decision analysis [49,50]. Hence, whether the proposed GDM approach can be utilized to discuss the CRP for LSGDM problems is worthy of discussion.

## Figures and Tables

**Table 1:** The number of the environmental emergencies occurred per year from 2012 to 2016, China.

	Year	2012	2013	2014	2015	2016
Times						
Total Number		542	712	471	334	304
Severe		5	3	3	3	3
Large		5	12	16	5	5
General		532	697	452	326	296

**Table 2:** Nine self-confidence language terms with its semantics.

Self-Confidence Language	Semantics
*s*_0_	None
*s*_1_	Very low
*s*_2_	Low
*s*_3_	Slightly low
*s*_4_	Medium
*s*_5_	Slightly high
*s*_6_	High
*s*_7_	Very high
*s*_8_	Prefect

**Table 3:** The detailed results of the $SCDL(e_{k})$ and $SCD(e_{k})$ of $e_{k}$ (k=1,2,3,4).

	Experts	$e_{1}$	$e_{2}$	$e_{3}$	$e_{4}$
*SCDL* and *SCD*					
$SCDL(e_{k})$		0.354	0.5	0.458	0.375
$SCD(e_{k})$		0.646	0.5	0.542	0.625

**Table 4:** The detailed results of the special case of the SC-FPRs (i=1,2,3,4).

${\tilde{P}}_{c}$	$SCS(x_{i})$	Rankings of Alternatives
$\left(\begin{array}{cccc} 0.5 & 0.35 & 0.5 & 0.43\\ 0.65 & 0.5 & 0.55 & 0.55\\ 0.5 & 0.4 & 0.5 & 0.55\\ 0.57 & 0.45 & 0.45 & 0.5 \end{array}\right)$	$\begin{array}{c} SCS(x_{1})=3.55\\ SCS(x_{2})=4.50\\ SCS(x_{3})=4.00\\ SCS(x_{4})=3.95 \end{array}$	$x_{2}≻x_{3}≻x_{4}≻x_{1}$

**Table 5:** Rankings of the alternatives with different values of *μ* and *τ*.

*μ* and *τ*	$SCS(x_{1})$	$SCS(x_{2})$	$SCS(x_{3})$	$SCS(x_{4})$	Rankings of Alternatives
μ=0,τ=1	2.1847	2.1971	2.3505	1.7678	$x_{3}≻x_{2}≻x_{1}≻x_{4}$
μ=0.1,τ=0.9	2.1868	2.1949	2.3504	1.7679	$x_{3}≻x_{2}≻x_{1}≻x_{4}$
μ=0.2,τ=0.8	2.1890	2.1927	2.3504	1.7680	$x_{3}≻x_{2}≻x_{1}≻x_{4}$
μ=0.3,τ=0.7	2.1912	2.1905	2.3503	1.7681	$x_{3}≻x_{1}≻x_{2}≻x_{4}$
μ=0.4,τ=0.6	2.1933	2.1882	2.3503	1.7682	$x_{3}≻x_{1}≻x_{2}≻x_{4}$
μ=0.5,τ=0.5	2.1838	2.1820	2.3399	1.7518	$x_{3}≻x_{1}≻x_{2}≻x_{4}$
μ=0.6,τ=0.4	2.1976	2.1838	2.3502	1.7684	$x_{3}≻x_{1}≻x_{2}≻x_{4}$
μ=0.7,τ=0.3	2.1998	2.1816	2.3501	1.7685	$x_{3}≻x_{1}≻x_{2}≻x_{4}$
μ=0.8,τ=0.2	2.2019	2.1794	2.3501	1.7686	$x_{3}≻x_{1}≻x_{2}≻x_{4}$
μ=0.9,τ=0.1	2.2041	2.1772	2.3500	1.7687	$x_{3}≻x_{1}≻x_{2}≻x_{4}$
μ=1,τ=0	2.2063	2.1750	2.3500	1.7688	$x_{3}≻x_{1}≻x_{2}≻x_{4}$

## Appendix A

**Table A1:** Symbol descriptions.

Notations	Descriptions
$X=\{x_{1},x_{2},\ldots ,x_{n}\}$	Finite set of environmental pollution emergency alternatives
$E=\{e_{1},e_{2},\ldots ,e_{m}\}$	Set of experts (decision makers)
$S^{SL}=\{s_{i}|i=0,1,\ldots ,g\}$	Set of the linguistic self-confidence of expert
$P_{k}={\left(p_{ij,k},l_{ij,k}\right)}_{n\times n}$	The SC-FPR of expert $e_{k}$
$\tilde{L}={\left({\tilde{l}}_{ij}\right)}_{n\times n}$	The self-confidence matrix of expert $e_{k}$
$SCDL(e_{k})$	Self-confidence deviation level of $e_{k}$
$SCD(e_{k})$	Self-confidence degree level of $e_{k}$
$w^{sub}=(w_{1}^{sub},w_{2}^{sub},\ldots ,w_{m}^{sub})$	Subjective weight set of expert
$w^{obj}=(w_{1}^{obj},w_{2}^{obj}\ldots w_{m}^{obj})$	Objective weight set of expert
$w=(w_{1},w_{2},\ldots ,w_{k})$	Decision weight set of expert
μ and τ	The parameters to control the weight between subjective and objective weights of expert
$P_{c}={\left(p_{ij,c},l_{ij,c}\right)}_{n\times n}$	The SC-FPR of collective
$SCS(x_{i})$	The self-confidence score function of $x_{i}$
